# Targeting gut–liver–kidney axis: microbiota-derived metabolites and therapeutic implications

**DOI:** 10.1186/s12964-025-02625-x

**Published:** 2026-01-14

**Authors:** Yufei Zhang, Cuiting Sun, Yudian Wang, Haojun Zhang, Yuyan Fan, Hailing Zhao, Ping Li

**Affiliations:** 1 College of Traditional Chinese Medicine, Hubei University of Chinese Medicine, Wuhan, Hubei China; 2 China-Japan Friendship Hospital, Capital Medical University, Beijing, China; 3 The Department of Traditional Chinese Medicine, Beijing Tiantan Hospital, Capital Medical University, Beijing, China; 4 Institute of Clinical Medical Sciences, China-Japan Friendship Hospital, Beijing, China

**Keywords:** Gut–liver–kidney axis, Microbial metabolites, Short-chain fatty acids, Trimethylamine-N-Oxide, Therapeutic strategies

## Abstract

The gut–liver–kidney axis has emerged as a central regulatory network orchestrating metabolic, immune, and inflammatory homeostasis across organ systems. At its core lies the dynamic interplay between gut microbiota and host metabolism. Dysbiosis and impaired intestinal barrier integrity facilitate the systemic translocation of microbial metabolites—such as short-chain fatty acids (SCFAs), bile acids (BAs), trimethylamine-N-oxide (TMAO), and tryptophan derivatives—which profoundly influence hepatic lipid metabolism, renal immune responses, and overall metabolic balance. This review examines the molecular mechanisms through which gut-derived metabolites contribute to liver and kidney pathology, emphasizing inter-organ signaling and the pathological cascade of the “leaky gut–hepatic injury–renal dysfunction” loop. We critically evaluate emerging therapeutic strategies targeting this axis, including probiotic supplementation, fecal microbiota transplantation (FMT), dietary modulation (low-protein, high-fiber regimens), and pharmacological detoxification (e.g., AST‑120, molecular adsorbent recirculating systems [MARS]). Finally, we propose a conceptual “diet–microbiota–drug” triad to guide precision interventions, and discuss current challenges such as interindividual variability, the lack of standardized assessment tools, and the need for integrative multi‑omics and clinical validation. A deeper mechanistic understanding of gut–organ crosstalk may pave the way for innovative therapies to restore systemic metabolic homeostasis.

## Introduction

The human body’s organs operate in concert to sustain systemic metabolic balance. Among these inter-organ networks, the gut–liver–kidney axis has recently garnered increasing attention for its central role in metabolic regulation and chronic disease progression. Epidemiological studies indicate that patients with metabolic dysfunction–associated steatotic liver disease (MASLD)—a term that has recently replaced the former classifications of non-alcoholic fatty liver disease (NAFLD) and metabolic dysfunction–associated fatty liver disease (MAFLD)—have a nearly 40% higher long-term risk of developing chronic kidney disease (CKD) [1]. Research has revealed that gut dysbiosis-induced metabolic toxins, via portal circulation and systemic inflammatory responses, drive hepatic lipid metabolism dysregulation and renal fibrosis progression, underscoring the critical interplay among the gut, liver, and kidneys in metabolic disorders [2–4]. This concept, originally conceptualized as an expansion of the gut–liver and gut–kidney axes, has evolved into an important framework for understanding multi-organ metabolic disorders [5, 6]. This axis is expected to become a frontier in metabolic and chronic inflammatory disease research, providing a theoretical basis for explaining the synergistic progression of multi-organ pathologies. The imbalance of this axis may help explain the co-morbidity of metabolic diseases, such as diabetes mellitus and MASLD, with CKD, but also provides a new perspective on cross-organ targeting of therapies [7]. This convergence has brought the gut–liver–kidney axis to the forefront of metabolic disease research.

The gut-liver-kidney axis maintains systemic metabolic homeostasis through bidirectional interactions among these organs. Central to this axis is the integrity of the intestinal barrier, which prevents translocation of pathogenic microbes and harmful metabolites [8]. For instance, the liver reciprocally regulates gut microbiota composition and facilitates barrier repair through bile acids (BAs) synthesis [9]. Concurrently, the kidneys maintain metabolic equilibrium by clearing gut- and liver-derived uremic toxins, modulating fluid–electrolyte balance to prevent excessive accumulation of these toxins, which could compromise intestinal barrier integrity [10–12]. Furthermore, these organs coordinate via metabolite signaling pathways such as bile acid–farnesoid X receptor (FXR) axis and short-chain fatty acids (SCFAs)-G protein-coupled receptor 43 (GPR43) pathway and immune mediators, collectively forming an integrated network for metabolite transport, inflammation regulation, and barrier homeostasis [13–15]. When gut dysbiosis or barrier dysfunction occurs, microbial metabolites such as SCFAs and endotoxins enter the liver via the portal circulation, disrupting hepatic detoxification and metabolic processes [3]. Simultaneously, renal accumulation of uremic toxins exacerbates oxidative stress and fibrosis, while renal impairment further destabilizes gut microbiota [10]. Consequently, dysregulated inter-organ communication triggers metabolic disturbances and a pathological cascade of multi-organ damage. Once disrupted, this axis contributes to hepatic inflammation, renal fibrosis, and systemic metabolic dysfunction.

**Fig. 1:**
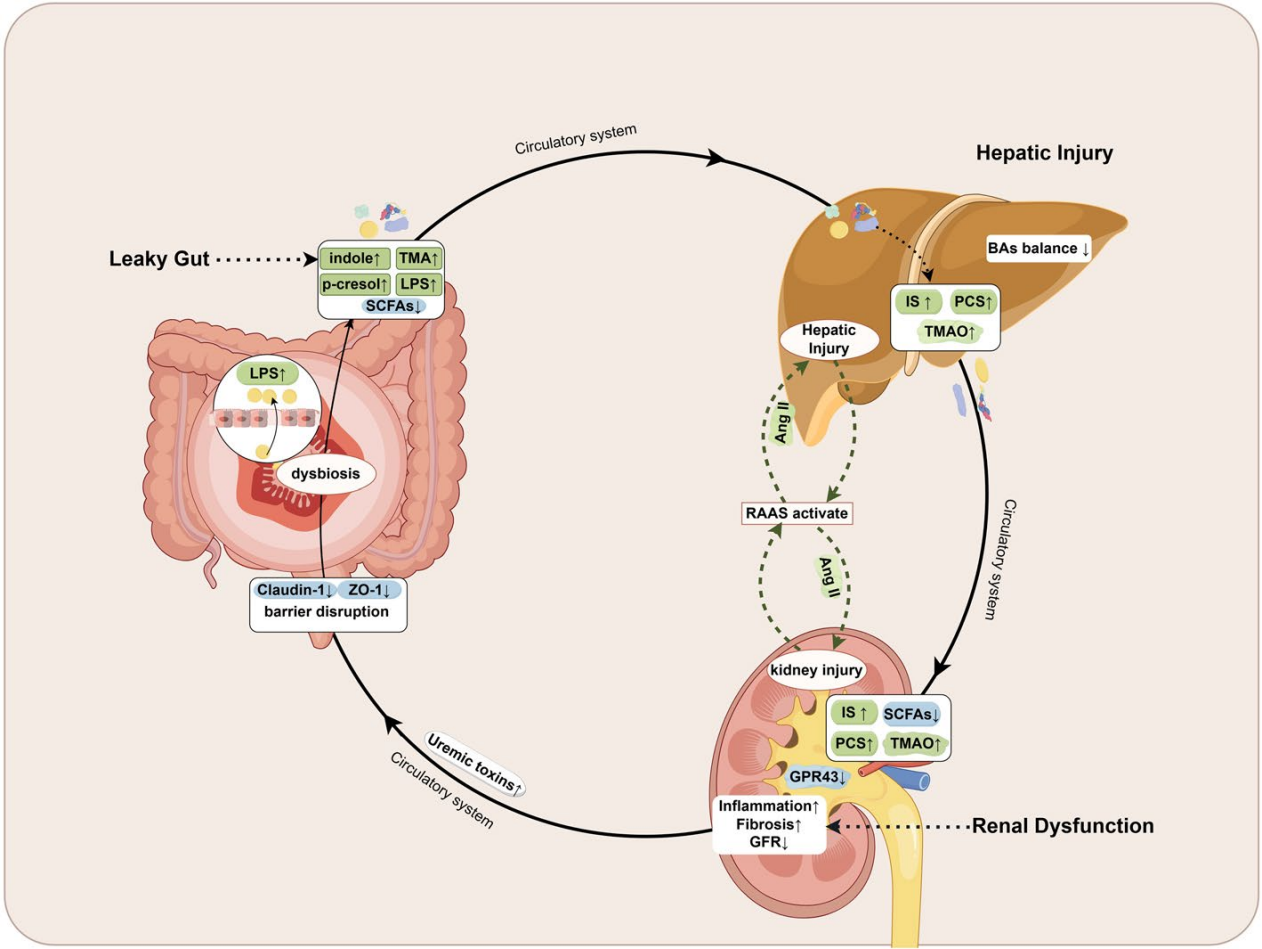
Crosstalk within the gut–liver–kidney axis under pathological conditions. Intestinal dysbiosis and barrier disruption facilitate translocation of gut-derived microbial products (including endotoxins and uremic precursors) into the portal circulation. These metabolites reach the liver, where they activate inflammatory signaling pathways, disrupt BAs homeostasis, and contribute to hepatic injury. Meanwhile, reduced levels of SCFAs exacerbate both hepatic and renal damage by impairing anti-inflammatory signaling. In particular, decreased activation of GPR43 in the kidney promotes inflammation and fibrosis, aggravating renal injury. Hepatic and renal dysfunction synergistically activate the RAAS, promoting the release of pro-inflammatory mediators such as angiotensin II (Ang II), which further aggravate organ injury. In parallel, microbially derived uremic toxins—such as IS, PCS, and TMAO—enter systemic circulation and contribute to renal inflammation, interstitial fibrosis, and a decline in glomerular filtration rate. Conversely, hepatic and renal dysfunction lead to systemic accumulation of uremic toxins, which in turn impair gut epithelial integrity and increase intestinal permeability. This establishes a self-perpetuating cycle linking gut barrier disruption, hepatic injury, and progressive renal dysfunction

**Fig. 2:**
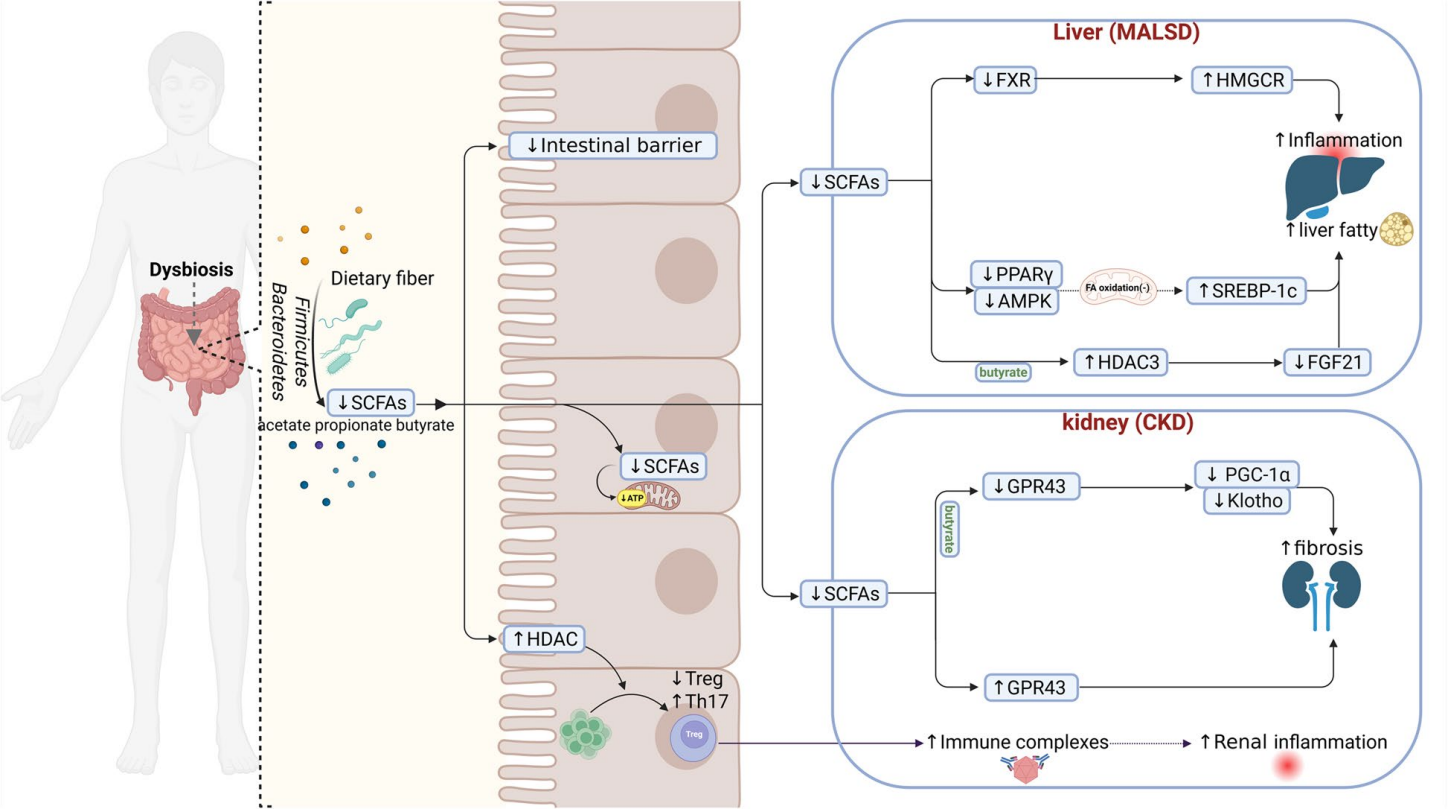
Mechanistic pathways through which SCFAs regulate the gut–liver–kidney axis under dysbiotic conditions. Gut dysbiosis reduces the abundance of SCFA-producing bacteria from the *Firmicutes* and *Bacteroidetes* phyla, leading to decreased levels of SCFAs, including acetate, propionate, and butyrate. This reduction compromises intestinal barrier integrity by downregulating tight junction proteins. In the gut mucosa, SCFA deficiency impairs regulatory T cell (Treg) differentiation, promotes Th17-mediated proinflammatory responses, and facilitates immune complex formation, thereby contributing to renal inflammation. In the kidney, diminished butyrate availability reduces GPR43 activation, downregulates Klotho and PGC-1α expression, and exacerbates inflammation and fibrosis. In the liver, insufficient SCFAs fail to adequately activate FXR and peroxisome proliferator–activated receptor gamma, resulting in increased HMG-CoA reductase (HMGCR) activity, hepatic steatosis, and inflammation. The suppression of butyrate-induced AMPK signaling also enhances SREBP-1c–mediated lipogenesis and reduces FGF21 expression via inadequate histone deacetylase 3 inhibition. Collectively, these alterations drive the progressive deterioration of gut–liver–kidney axis homeostasis

**Fig. 3:**
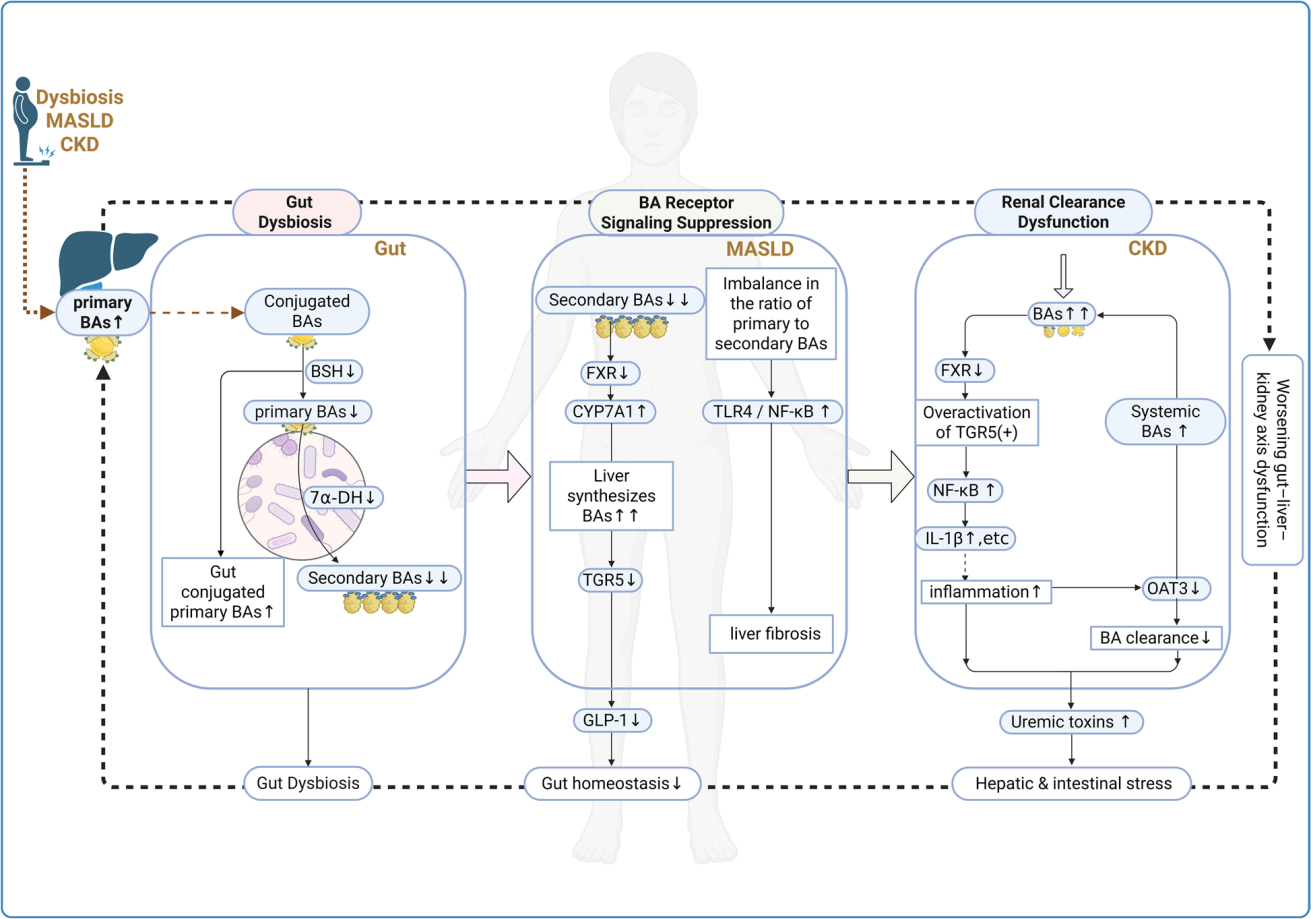
Disruption of bile acid metabolism along the gut–liver–kidney axis under pathological conditions. Gut microbial dysbiosis leads to decreased activity of BSH and 7α-dehydroxylase (7α-DH), resulting in impaired conversion of conjugated primary BAs into secondary BAs. An altered primary/secondary bile-acid ratio perturbs BA-receptor signaling, disrupting enterohepatic homeostasis and promoting hepatic and renal inflammation. In the kidney, Excessive BAs may dysregulate FXR and TGR5 signaling, thereby enhancing NF-κB pathway activation in renal tubular cells. This promotes the release of proinflammatory cytokines such as IL‑6, TNF‑α, and MCP‑1, thereby exacerbating renal interstitial inflammation. Concurrently, impaired renal clearance due to downregulation of organic anion transporter 3 (OAT3) results in systemic BA accumulation and contributes to the retention of uremic toxins. These alterations form a self-reinforcing cycle that exacerbates gut–liver–kidney axis dysfunction

**Fig. 4:**
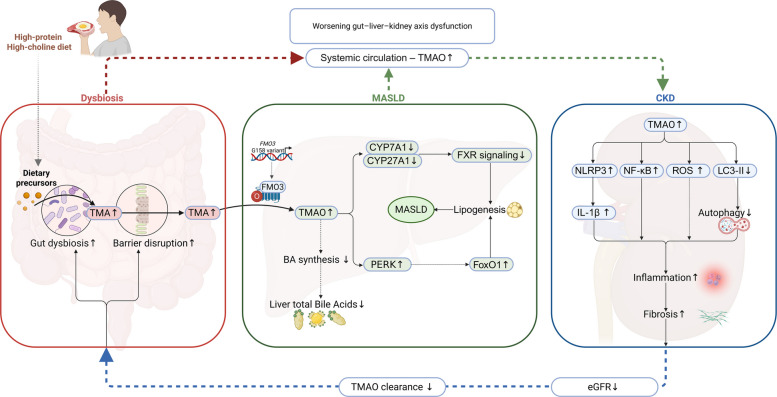
TMAO-mediated pathophysiological cascade across the gut–liver–kidney axis. Dietary choline, phosphatidylcholine, and L‑carnitine are metabolized by gut microbiota into TMA, a process enhanced by gut dysbiosis in CKD, and increased TMA generation contributes to intestinal barrier disruption. TMA enters the liver via the portal vein and is oxidized by FMO3 into TMAO. Elevated TMAO levels suppress FXR signaling and downregulate CYP7A1/CYP27A1, reducing bile acid synthesis, while activating the PERK–FoxO1 axis to promote hepatic lipogenesis. Circulating TMAO stimulates renal NLRP3 inflammasome activation, increases proinflammatory cytokines (interleukin‑1 beta, IL-1β; interleukin‑18, IL‑18), NF-κB signaling, and reactive oxygen species, and reduce LC3-II, thereby inhibiting autophagy cumulatively driving renal interstitial inflammation and fibrosis. Declining glomerular filtration in CKD impairs TMAO clearance, forming a feedback loop that exacerbates systemic TMAO accumulation and multi-organ injury

Despite growing mechanistic insights, most existing studies have focused on isolated or pairwise organ interactions, with limited integration of tripartite crosstalk. This review synthesizes emerging evidence on how microbial metabolites shape inter-organ communication within the gut–liver–kidney axis. We further evaluate translational bottlenecks and propose an integrated “diet–microbiota–drug” framework to advance precision medicine in this domain. To guide the reader through the complex interactions within the gut–liver–kidney axis, this review uses four schematic figures as a narrative thread. Figure 1 outlines the integrated pathophysiological framework and the self-reinforcing loops linking the three organs. Figures 2, 3 and 4 summarize the organ-specific and systemic actions of major microbial metabolites and illustrate how these signals converge on shared mechanistic nodes relevant to disease progression. For clarity, Figs. 1, 2, 3 and 4 are intended to be read as an integrated visual roadmap: Fig. 1 frames the central pathological loops of the axis, while Figs. 2, 3 and 4 map individual metabolite classes (SCFAs, BAs, Trimethylamine‑N‑Oxide [TMAO] and related compounds) onto the mechanistic nodes illustrated in Fig. 1.

### The gut-liver-kidney axis

#### Gut–liver axis: metabolic and immune interactions

The gut–liver axis is anatomically and functionally connected via the portal vein, which delivers approximately 75% of the liver’s blood supply and enables the direct transfer of gut-derived metabolites, including microbial products and endotoxins, to the liver [16–18]. The intestinal mucosal barrier—comprising tight junction proteins, a mucus layer, and immune cells—plays a central role in maintaining compartmentalization and supporting gut–liver axis integrity [19–21]. In turn, the liver regulates intestinal homeostasis through bile secretion and immune mediators such as immunoglobulin A (IgA), which inhibit the proliferation of pathogenic bacteria [22]. Microbial SCFAs, produced from dietary fiber fermentation, reach the liver via the portal circulation. In the liver, they modulate hepatocyte energy metabolism and inflammatory signaling [23, 24]. Butyrate, in particular, enhances mitochondrial function by activating AMP‑activated protein kinase (AMPK), thereby attenuating hepatic steatosis [25]. Conversely, liver-derived BAs, along with their primary and secondary metabolites, regulate gut microbiota composition by activating FXR and G protein–coupled bile acid receptor 1 (TGR5), which are key pathways for maintaining mucosal immunity and intestinal barrier integrity [26–28]. BAs act as signaling molecules targeting nuclear receptors (FXR) and membrane receptors (TGR5) to coordinate hepatic synthesis and metabolic homeostasis, linking gut microbial activity to liver function [29, 30].

The intestinal barrier includes both physical and immune components. Physical structures such as the mucus layer, epithelial cells, tight junctions, and the gut vascular barrier together form the anatomical interface between the gut and liver. These barriers limit microbial and toxin translocation while allowing nutrient absorption for hepatic processing [17, 31]. Disruption of the gut–liver axis impairs barrier integrity, leading to endotoxin translocation and promoting liver injury [32]. For example, lipopolysaccharide (LPS) transported via the portal vein activates Toll-like receptor 4 (TLR4)/nuclear factor-κB (NF-κB) signaling in Kupffer cells, thereby initiating pro-inflammatory cascades and fibrotic remodeling [33]. Clinically, elevated serum levels of LPS-binding protein correlate with liver damage severity in patients with MASLD [34]. Gut dysbiosis further exacerbates this axis by disrupting bile acid homeostasis, contributing to the development of MASLD and cholestatic liver diseases [35, 36]. Under dysbiotic conditions, commensal bacteria such as Bacteroides spp. mediate bile acid deconjugation via bile salt hydrolase (BSH), which is a prerequisite for the subsequent 7α-dehydroxylation by other gut microbes to generate secondary BAs [37, 38]. Reduced BSH activity impairs enterohepatic recycling and reduces secondary BA production, and increases intestinal levels of unmetabolized conjugated BAs. This imbalance disrupts FXR and TGR5 signaling, exacerbating metabolic dysfunctions such as insulin resistance, hepatic steatosis, and dyslipidemia [39–41].

#### Gut–kidney axis: microbial toxins and renal responses

The gut–kidney axis mediates bidirectional communication through microbial metabolites, uremic toxins, and immune signaling pathways [42]. Under physiological conditions, the gut microbiota metabolizes tryptophan into indole derivatives, some of which are excreted by the kidneys and help regulate oxidative stress in renal tubular epithelial cells [42]. For example, indole-3-propionic acid (IPA) suppresses IS-induced expression of fibrotic and inflammatory genes in proximal tubular cells [43]. SCFAs produced by the gut microbiota not only enhance the expression of zonula occludens-1 (ZO-1) and claudin-1 in the gut but also confer renal protection. Butyric acid, produced by *Faecalibacterium spp*., and acetic acid, produced by Bifidobacterium, enhance epithelial barrier integrity by activating GPR43. This upregulates ZO-1 and claudin-1 in renal tubular epithelial cells, thereby preventing epithelial apoptosis [44, 45].

Disruption of the gut–kidney axis increases intestinal permeability, facilitating the absorption of uremic toxin precursors such as indole, p-cresol, and trimethylamine (TMA) into the circulation [46]. These compounds are subsequently metabolized by the liver into indoxyl sulfate (IS), p-cresyl sulfate (PCS), and TMAO [47]. In CKD, reduced glomerular filtration and impaired tubular secretion result in toxin accumulation, which directly injures renal tubular epithelial cells [48]**.** IS and PCS promote interstitial fibrosis, while TMAO contributes to glomerular injury and vascular inflammation [48, 49]. This establishes a self-perpetuating cycle that accelerates decline and tubular dysfunction. Additionally, accumulated urea can diffuse back into the colon and be metabolized into ammonia by urease-producing bacteria, further disrupting the intestinal barrier, promoting dysbiosis and endotoxemia, and reinforcing the “leaky gut–kidney injury” loop [50, 51]. Clinical studies have revealed significant alterations in the gut microbiota of CKD patients, including decreased abundance of SCFA-producing bacteria and an overgrowth of *Ruminococcus* species such as R. gnavus [52–54]. This dysbiotic state correlates with increased intestinal permeability and accelerated renal fibrosis [4]. Secretory IgA, a key regulator of gut immunity, also engages in bidirectional communication with the renal innate immune system. Microbial dysbiosis enhances macrophage infiltration in the kidney and activates the transforming growth factor-β1 (TGF-β1) signaling pathway, thereby exacerbating renal inflammation and fibrosis [55, 56].

#### Liver–kidney axis: hemodynamic, metabolic, and inflammatory crosstalk

The anatomical foundation of the liver–kidney axis lies in the redistribution of blood flow induced by elevated sinusoidal resistance during portal hypertension. Functionally, it is sustained by reciprocal metabolic and endocrine interactions between the two organs [57]. Metabolically, the liver detoxifies ammonia via the urea cycle, whereas the kidneys excrete the resulting urea [58]. In chronic liver disease, the kidneys partially compensate for hepatic metabolic dysfunction by upregulating gluconeogenesis [58]. However, severe hepatic dysfunction leads to urea cycle failure and consequent hyperammonemia. Although the kidneys continue to excrete urea, reduced hepatic detoxification capacity compromises systemic ammonia clearance [59]. Furthermore, metabolic acidosis secondary to renal dysfunction inhibits glutamine synthetase activity, promoting ammonia overproduction in renal tubules [60]. Together, these processes establish a vicious cycle involving hepatic detoxification failure, renal compensatory overload, and acidosis-driven inhibition of hepatic enzymatic activity. The renin–angiotensin–aldosterone system (RAAS) plays a central role in the pathogenesis of liver and kidney diseases. Angiotensin II (Ang II), the primary RAAS effector, contributes to multi-organ dysfunction by activating its type 1 receptor. Hemodynamically, Ang II induces vasoconstriction in hepatic sinusoids and glomerular afferent arterioles, aggravating portal hypertension and reducing glomerular filtration rate [61–63]. Profibrotically, Ang II activates the TGF-β1 pathway, promoting both hepatic stellate cell activation and renal interstitial fibrosis [64, 65]. Endocrinologically, Ang II stimulates aldosterone secretion, enhancing renal sodium and water retention and thereby exacerbating ascites in cirrhotic patients [66]. Thus, aberrant RAAS activation serves as a molecular nexus in the development of hepatorenal syndrome with acute kidney injury in cirrhosis and hepatic metabolic disturbances in CKD. In parallel, hepatic mitochondrial dysfunction during cirrhosis leads to increased reactive oxygen species production and reduced nitric oxide availability, forming a feedback loop that impairs mitochondrial energy metabolism in renal tubular cells [67, 68].

The frequent coexistence of MASLD and CKD further underscores the presence of shared metabolic regulatory disruptions. For instance, bile-acid dyshomeostasis perturbs BA-receptor signaling, contributing to proinflammatory responses that affect both liver and kidney [69, 70]. Hepatorenal syndrome exemplifies this pathological crosstalk, where portal hypertension–induced hemodynamic alterations and systemic inflammation act synergistically to drive organ dysfunction.

#### Integrated pathological framework: three core mechanisms driving axis dysregulation

As summarized in Fig. 1, the gut–liver–kidney axis operates as an integrated network in which gut barrier disruption, hepatic metabolic dysregulation, and renal dysfunction form self-reinforcing loops. Accordingly, Fig. 1 serves as the schematic backbone for the mechanistic discussion that follows. Dysregulation of the gut–liver–kidney axis is marked by a self-reinforcing pathological loop involving “leaky gut–hepatic injury–renal dysfunction.” As illustrated in Fig. 1, gut-derived metabolites enter the liver via the portal vein, initiating a cascade of metabolic and inflammatory signaling events that extend along the liver–kidney axis. First, gut microbiota dysbiosis contributes to hepatic injury. For example, an imbalanced Firmicutes-to-Bacteroidetes ratio disrupts tight junction integrity and increases intestinal permeability, promoting the translocation of endotoxins and uremic toxins into the circulation [71, 72]. Second, the liver responds to gut-derived microbial products (e.g., endotoxins) by activating innate immune and metabolic responses that amplify systemic low-grade inflammation [73]. Third, these inflammatory mediators impair renal tubular epithelial cell function, inhibit regeneration, and promote fibrotic remodeling [74]. Finally, renal failure leads to the accumulation of nitrogenous waste products such as urea. These are converted by the gut microbiota into toxic compounds like ammonia, further compromising intestinal barrier integrity [72]. Clinical studies report increased markers of endotoxemia in end-stage renal disease, consistent with gut barrier impairment and systemic exposure to gut-derived microbial products [42]. This vicious cycle is particularly prominent in metabolic disorders such as diabetic kidney disease. Under hyperglycemic conditions, the growth of butyrate-producing bacteria (e.g., *Faecalibacterium prausnitzii*) is selectively suppressed, leading to a reduction in SCFA levels. The consequent attenuation of histone deacetylase (HDAC) inhibition disrupts the proliferator–activated receptor alpha/fibroblast growth factor 21 (PPARα/FGF21) signaling axis and promotes hepatic lipid accumulation and renal interstitial fibrosis in diabetic kidney disease [75–80]. Accordingly, three core pathological mechanisms underlie gut–liver–kidney axis dysfunction: gut microbiota imbalance, compromised barrier integrity, and the accumulation of toxic metabolic products.

Figure 1 synthesizes the principal pathological loops linking gut barrier failure, hepatic metabolic injury, and renal dysfunction. This integrated framework highlights how disturbances originating in one organ propagate across the gut–liver–kidney axis and provides the foundation for understanding the systemic actions of microbial metabolites.

### Gut-derived metabolites and the gut–liver–kidney axis

Microbial metabolites act through interconnected pathways rather than isolated mechanisms within the gut–liver–kidney axis. Although each metabolite class has distinct functions, many converge on shared regulatory nodes such as barrier integrity, nuclear receptors, inflammation, and fibrosis. The following subsections describe individual metabolites while emphasizing their complementary or opposing roles in shaping axis homeostasis. Figures 2, 3 and 4 then map the organ-specific and systemic actions of major metabolite classes (SCFAs, BAs, and TMAO, respectively) onto these shared nodes.

#### SCFAs

The diverse protective actions of SCFAs across the gut–liver–kidney axis are summarized in Fig. 2, which links SCFA-dependent pathways (barrier maintenance, immune regulation, FXR/AMPK signaling) to downstream hepatic and renal outcomes. SCFAs are key metabolites produced by gut microbial fermentation of dietary fiber, with acetate, propionate, and butyrate being the primary components [81]. These are mainly synthesized by anaerobic gut microbes, particularly members of the Firmicutes and Bacteroidetes phyla. Notable producers include *Faecalibacterium prausnitzii*, *Roseburia* spp., *Eubacterium rectale*, *Anaerobutyricum (Eubacterium) hallii*, and *Coprococcus* spp. [82]. As critical signaling molecules in the gut–liver–kidney axis, SCFAs act on both the liver and kidneys via portal and systemic circulation, influencing metabolic regulation, immune modulation, and inflammation control [83]. Altered SCFA profiles are closely associated with gut–liver–kidney axis–related metabolic disorders. In the liver, SCFAs regulate hepatocyte metabolism and inflammation via the portal vein. Butyrate activates the AMP-activated protein kinase pathway and upregulates peroxisome proliferator–activated receptor gamma, enhancing mitochondrial fatty acid oxidation and inhibiting sterol regulatory element-binding protein-1c (SREBP-1c)–mediated lipogenesis. Additionally, butyrate stimulates fibroblast growth factor 21 (FGF21) expression by inhibiting histone deacetylase 3, thereby reducing hepatic lipid accumulation and improving MASLD [84, 85]. SCFAs also influence FXR signaling in the intestine and liver, improving bile acid metabolism, suppressing the cholesterol biosynthesis–related gene HMG-CoA reductase, lowering cholesterol levels, and mitigating liver injury and ileal inflammation [86, 87]. Among SCFAs, butyrate enhances intestinal barrier integrity by upregulating tight junction proteins (e.g., occludin and ZO-1), thereby reducing translocation of gut-derived endotoxins and downstream hepatic inflammation [88]. Propionate mitigates hepatocyte apoptosis under oxidative stress by scavenging reactive oxygen species and inducing the antioxidant enzyme superoxide dismutase [89].

Emerging evidence indicates that SCFAs also shape mucosal immunity by regulating innate lymphoid cell type 3 (ILC3) function. SCFAs promote ILC3-mediated secretion of interleukin-22, a cytokine that drives epithelial proliferation, antimicrobial peptide production, and mucosal repair [90–92]. Through this SCFA–ILC3–interleukin-22 axis, dietary fiber–derived metabolites reinforce intestinal barrier integrity and restrict translocation of gut-derived microbial products to the liver—thereby providing an additional immunological mechanism supporting the gut–liver–kidney axis [93, 94].

In the kidneys, SCFAs reach target sites via systemic circulation and influence renal tubular function and the immune microenvironment [95]. SCFAs confer renoprotective effects primarily through two mechanisms: (1) enhancing regulatory T cell differentiation via HDAC inhibition in the gut immune microenvironment, thereby reducing immune complex–mediated glomerulonephritis; and (2) acting directly on podocytes and tubular epithelial cells via G protein–coupled receptor–dependent pathways, which alleviate oxidative stress and inflammation in glomerular mesangial cells, ultimately reducing hyperglycemia-induced renal injury and fibrosis [15, 44, 96–98]. Butyrate also maintains expression of protective renal factors such as Klotho and proliferator-activated receptor gamma coactivator 1-alpha (PGC-1α) and improves kidney function through GPR43 signaling, thereby attenuating fibrosis [99]. In acute kidney injury, SCFAs inhibit the NF-κB signaling pathway, reducing systemic and local cytokine release and improving renal function [99]. In CKD, intestinal dysbiosis leads to reduced SCFA production, which promotes uremic toxin accumulation and compromises barrier integrity, contributing to a vicious gut–kidney cycle [100, 101]. Animal studies demonstrate that exogenous butyrate supplementation restores gut microbiota diversity, lowers serum creatinine and blood urea nitrogen, and ameliorates kidney function in CKD models [102]. Clinical studies also show that serum butyrate levels positively correlate with estimated glomerular filtration rate (eGFR) in CKD patients [103].

Collectively, SCFAs exert coordinated effects on the liver and kidneys through portal and systemic circulation, establishing a tripartite regulatory network encompassing metabolism, immunity, and barrier integrity (Fig. 2). Core mechanisms include FXR/GPR signaling modulation, suppression of toxic metabolite accumulation, and restoration of mitochondrial function [104]. Despite growing evidence of hepatorenal protection by SCFAs, several challenges remain. First, individual SCFAs exhibit functional heterogeneity across the gut–liver–kidney axis [105–107]. Second, the safety of SCFA-based therapies requires further investigation; high oral doses have been linked to ureteral inflammation and hydronephrosis [108]. Advanced techniques such as single-cell sequencing and metabolomics may help elucidate tissue-specific and dose-dependent SCFA effects across organs, especially in kidney diseases.

#### Bile acids

Figure 3 depicts how dysregulation of bile acid metabolism alters FXR/TGR5 signaling and links intestinal BA transformations to hepatic and renal inflammation and fibrosis. BAs are cholesterol-derived metabolites synthesized in the liver as primary BAs, mainly cholic acid (CA) and chenodeoxycholic acid (CDCA). These primary BAs are subsequently conjugated with glycine or taurine to form conjugated BAs, such as glycocholic acid and taurochenodeoxycholic acid, and are then secreted into the intestine via bile flow [109]. In the intestinal lumen, gut microbiota initiate BA metabolism by producing BSH enzymes, which deconjugate glycine- or taurine-bound BAs, generating free primary BAs again. This enzymatic activity is widespread among anaerobic commensals such as *Lactobacillus*, *Bifidobacterium*, *Clostridium*, and *Bacteroides* species [110, 111]. A portion of these free primary BAs is further converted into secondary BAs via 7α-dehydroxylation, primarily by *Clostridium scindens*; for instance, CA and CDCA are converted into deoxycholic acid (DCA) and lithocholic acid, respectively [109, 112]. Approximately 95% of total BAs—both primary and secondary—are reabsorbed in the distal ileum and returned to the liver via the portal vein, completing the enterohepatic circulation, a key regulatory loop in gut–liver homeostasis [112].

Bile acids signal through nuclear and membrane receptors—most notably FXR and TGR5—to integrate metabolic and immune homeostasis across the gut–liver axis [113, 114]. FXR-mediated bile-acid signaling regulates hepatic BA synthesis and metabolic-inflammation programs, thereby maintaining lipid and glucose homeostasis [113, 115]. TGR5, as a membrane-bound G protein–coupled receptor, promotes thermogenesis in brown adipose tissue by upregulating uncoupling protein 1, stimulates intestinal secretion of glucagon-like peptide-1 to improve glucose homeostasis, and inhibits NF-κB–mediated proinflammatory cytokine production by immune cells [114, 116, 117]. Together, FXR and TGR5 maintain lipid and glucose metabolism as well as immune-inflammatory homeostasis by coordinating BA pool composition and receptor-mediated signaling.

Under pathological conditions, gut microbiota dysbiosis disrupts the ratio of primary to secondary BAs. This imbalance is often associated with a decreased abundance of BA-converting bacteria such as *Clostridium scindens* and increased levels of *Bacteroides* spp. [118]. Elevated DCA levels can indirectly activate the TLR4/NF-κB pathway in hepatic stellate cells, exacerbating liver fibrosis. Concurrently, excessive circulating BAs induce mitochondrial oxidative stress in renal tubular epithelial cells, accelerating CKD progression [39, 119]. Hydrophobic bile-acid accumulation perturbs BA-receptor signaling in hepatic and renal cells, leading to activation of downstream proinflammatory pathways and recruitment of inflammatory cells [120]. Notably, BA concentrations are significantly increased in CKD patients, possibly due to impaired function of organic anion transporter 3 in the kidney. This dysfunction impedes the excretion of BAs and uremic toxins, promoting their systemic accumulation and contributing to metabolic dysregulation along the gut–liver–kidney axis, ultimately establishing a vicious cycle [6] (Fig. 3).

#### Tryptophan-derived metabolites

Tryptophan, an essential amino acid obtained primarily from the diet, is metabolized through two main pathways: the host-driven kynurenine pathway and the microbiota-dependent indole pathway. Together, these pathways regulate immune and metabolic homeostasis within the gut–liver–kidney axis [121, 122]. In the host pathway, inflammatory stimuli induce the expression of indoleamine 2,3-dioxygenase 1 (IDO1) in hepatocytes and immune cells, catalyzing the conversion of tryptophan into kynurenine. Kynurenine and its downstream metabolites, such as quinolinic acid, activate the aryl hydrocarbon receptor (AhR), thereby suppressing proinflammatory signaling and inhibiting hepatic stellate cell activation, which slows the progression of liver fibrosis [123]. In the microbial pathway, approximately 5% of dietary tryptophan is converted by gut microbiota into various indole derivatives. Most indole is further sulfated in hepatocytes by the sulfotransferase SULT1A1 to form IS, while a smaller fraction is transformed into IPA via the indolepyruvate pathway by specific commensals, including *Clostridium sporogenes* [124, 125].

Although both IS and IPA activate AhR, they exert divergent downstream effects within the gut–liver–kidney axis. IS, a well-characterized uremic toxin, accumulates in CKD and induces oxidative stress and mitochondrial dysfunction in renal tubular epithelial cells. Elevated IS levels correlate with increased abundance of indole-producing bacteria, particularly *Escherichia coli* [126]. In contrast, IPA exerts anti-inflammatory and hepatoprotective effects. It reduces endotoxemia and suppresses NF-κB signaling, thereby ameliorating hepatic inflammation and steatosis in patients with MASLD. Furthermore, IPA enhances intestinal barrier integrity and limits endotoxin translocation, indirectly supporting liver and kidney function [125, 127, 128]. Clinical metabolomic studies have shown that reduced serum IPA concentrations in patients with liver cirrhosis are significantly associated with decreased gut microbial diversity. These findings suggest that the microbiota–metabolite axis may serve as an early biomarker for liver–kidney injury [122, 127]. Notably, the balance of tryptophan metabolism is tightly regulated by host–microbiota interactions. Probiotic strains such as *Lactobacillus reuteri* and *Bifidobacterium* spp. have been shown to enhance IPA production, whereas high-protein diets and gut dysbiosis are associated with increased IS accumulation in both clinical and preclinical studies [129–132]. Accordingly, emerging therapeutic strategies aimed at restoring gut–liver–kidney axis homeostasis include supplementation with IPA precursors, administration of specific probiotic strains, inhibition of IDO1, and adsorption of IS.

#### TMAO

The multi-organ pathological cascade driven by TMAO is summarized in Fig. 4. TMAO is a key gut microbiota–derived metabolite implicated in the gut–liver–kidney axis. Dietary precursors such as choline, phosphatidylcholine, and L-carnitine are metabolized by intestinal microbes into TMA, which is subsequently transported via the portal vein to the liver and oxidized by flavin-containing monooxygenase 3 (FMO3) into TMAO [133, 134]. This metabolic process is influenced by both host genetics and dietary composition. Genetic polymorphisms in the FMO3 gene significantly affect circulating TMAO levels and renal function decline. For instance, a low-frequency allele at position 158 of FMO3 is associated with elevated plasma TMAO and accelerated reductions in eGFR. Additionally, diets rich in protein and choline substantially increase gut-derived TMAO levels [134, 135]. Once formed, TMAO enters systemic circulation and is primarily excreted via glomerular filtration and tubular secretion.

Clinical studies have demonstrated a positive correlation between plasma TMAO concentrations and the severity of MASLD [136]. TMAO contributes to hepatic steatosis by disrupting bile acid homeostasis and activating endoplasmic reticulum stress pathways. In animal models, TMAO reduces hepatic bile acid levels and inhibits the expression of key bile acid synthesis enzymes, including cholesterol 7α-hydroxylase (CYP7A1) and cytochrome P450 family 27 subfamily A member 1 (CYP27A1), thereby suppressing FXR signaling and promoting hepatic triglyceride accumulation [136, 137]. Furthermore, TMAO directly activates the endoplasmic reticulum stress sensor protein kinase RNA-like endoplasmic reticulum kinase (PERK), triggering PERK-mediated unfolded protein response signaling. This leads to the upregulation of transcription factors such as forkhead box protein O1 (FoxO1), which promote lipid biosynthesis, ultimately exacerbating hepatic lipogenesis [138]. Collectively, these findings suggest that TMAO exacerbates MASLD by suppressing bile acid synthesis and activating endoplasmic reticulum stress pathways, leading to intracellular lipid accumulation in hepatocytes.

In the kidney, TMAO accumulation is strongly associated with CKD progression. Reduced glomerular filtration in CKD impairs TMAO clearance, resulting in significantly elevated circulating levels [139]. Serum TMAO levels are inversely correlated with eGFR, indicating its potential utility as a biomarker for CKD. In a population-based cohort, TMAO exhibited moderate discriminatory ability for CKD, with an area under the receiver operating characteristic curve of 0.614 [140]. Experimental studies in animal and cellular models have shown that TMAO contributes to renal injury through multiple mechanisms. TMAO directly stimulates renal tubular epithelial cells, activates NOD-like receptor family pyrin domain-containing 3 (NLRP3), and promotes the release of proinflammatory cytokines such as IL-1β and IL-18, thereby exacerbating renal interstitial inflammation and fibrosis [141]. In a diabetic kidney disease rat model induced by a high-fat, high-sugar diet, TMAO supplementation worsened renal dysfunction and fibrosis, accompanied by increased expression of NLRP3, caspase-1, IL-1β, and IL-18 [141]. TMAO also disrupts mitochondrial function in tubular cells, downregulates autophagy-related proteins such as microtubule-associated proteins 1A/1B light chain 3 phosphatidylethanolamine conjugate (LC3-II) and autophagy-related protein 6, and promotes reactive oxygen species accumulation, leading to oxidative injury [142]. Although further research is warranted, current evidence indicates that TMAO activates the NF-κB pathway and induces chronic low-grade inflammation in the kidney, accelerating CKD progression (Fig. 4).

Within the gut–liver–kidney axis, CKD patients often exhibit gut microbiota dysbiosis. Impaired renal function reduces TMAO excretion, resulting in elevated plasma TMAO levels [136, 139]. Consequently, therapeutic strategies aimed at reducing TMAO production and accumulation are gaining attention. Dietary interventions—such as restricting red meat and other TMA precursors and increasing fiber intake—may suppress TMA-producing bacterial populations and lower TMAO levels [143]. Probiotic supplementation also holds promise. For example, *Lactobacillus rhamnosus* has been shown to significantly reduce plasma TMAO levels by inhibiting colonization by TMA-producing bacteria and enhancing gut barrier function, thereby reducing TMA absorption and hepatic conversion to TMAO [144]. However, TMAO also serves physiological roles, such as stabilizing protein structure and maintaining osmotic balance. Thus, interventions targeting TMAO must carefully balance its pathological effects with its essential biological functions [145].

#### Other metabolites

Branched-chain amino acids (BCAAs), including leucine, isoleucine, and valine, are essential amino acids whose metabolic imbalance is a hallmark of gut–liver–kidney axis disorders [146]. Within the gut–liver axis, the intestinal microbiota regulates BCAA synthesis and degradation, thereby modulating their systemic levels [147]. Dysbiosis—such as altered abundances of *Ileibacterium*, *Bifidobacterium*, and *Akkermansia*—may elevate plasma BCAA concentrations, activate the mammalian target of rapamycin signaling pathway, and upregulate lipogenic factors such as SREBP-1c, ultimately promoting hepatic lipogenesis and accelerating nonalcoholic fatty liver disease progression [148, 149]. However, multiple randomized controlled trials and meta-analyses have shown that BCAA supplementation can reduce the incidence of severe complications in patients with liver cirrhosis and improve clinical outcomes [150]. Within the gut–kidney axis, oral BCAA intake in diabetic nephropathy rat models significantly reduced renal oxidative stress and proteinuria, inhibited the JNK/TGF-β/MMP-9 pathway (c-Jun N-terminal kinase, JNK; matrix metalloproteinase-9, MMP-9), and alleviated early kidney injury [151]. In a mouse model of acute kidney injury, activation of the BCAA metabolic pathway enhanced mitochondrial function and reduced mechanistic target of rapamycin complex 1 activation, thereby protecting renal tubules and mitigating tissue damage [152]. These findings suggest that BCAAs can exert both protective and deleterious effects along the gut–liver–kidney axis, depending on metabolic context. Their biological impact is shaped by the type, concentration, and site-specific microenvironment of BCAA-derived metabolites [153]. Under intact metabolic conditions—characterized by stable gut microbiota, preserved mitochondrial function, and adequate enzymatic activity—BCAAs are catabolized into antioxidant and cytoprotective end products. In contrast, metabolic disturbances such as dysbiosis, age-related enzymatic decline, or mitochondrial dysfunction may lead to the accumulation of toxic intermediates, promoting oxidative stress and tissue injury. Thus, the physiological window for BCAA supplementation is highly individualized and context-dependent. For example, older adults with sarcopenia may benefit from moderate BCAA intake to support muscle protein synthesis and preserve lean mass [150]. Since leucine, isoleucine, and valine are typically ingested together and follow highly similar catabolic pathways, they are often studied as a group. Nonetheless, future investigations into the distinct physiological and molecular functions of individual BCAAs may help clarify their roles within the gut–liver–kidney axis [153].

Vitamin D metabolism also relies on the coordinated function of the gut–liver–kidney axis. The intestinal microbiota facilitates absorption of lipid-soluble vitamin D precursors (vitamin D₃/D₂) by enhancing fat absorption and preserving barrier integrity. The liver then converts vitamin D into 25-hydroxyvitamin D [25(OH)D], which is further hydroxylated in the kidney to its active form, 1,25-dihydroxyvitamin D [1,25(OH)₂D], via the enzyme 1α-hydroxylase [154–156]. Disruption of this conversion process is closely linked to the pathogenesis of MASLD and CKD–mineral and bone disorder [157]. Studies have reported a high prevalence of vitamin D deficiency in MASLD, with lower vitamin D levels correlating positively with the severity of hepatic fibrosis [158]. Vitamin D supplementation may suppress hepatic stellate cell activation, reduce expression of collagen and other fibrotic markers, and thereby attenuate liver fibrosis progression [159]. In CKD, impaired renal function reduces 1,25(OH)₂D synthesis, while gut microbiota dysbiosis further impairs vitamin D absorption. This dual impairment often results in deficiency, which contributes not only to CKD–mineral and bone disorder but also to secondary hyperparathyroidism and immune dysregulation [160, 161]. Probiotics such as *Lactobacillus rhamnosus* strain GG and *Lactobacillus plantarum* have been shown to activate vitamin D receptor (VDR) signaling in intestinal epithelial cells, suppressing inflammation [162]. However, the use of *Lactobacillus rhamnosus GG* requires caution, as a study in a rat model of CKD revealed that it exacerbates vascular calcification in chronic kidney disease [163]. VDR activation may also stabilize tight junction proteins and strengthen intestinal barrier function, thereby potentially reducing oxidative damage to the liver and kidneys caused by endotoxin translocation [164].

High dietary fructose intake has emerged as a relevant metabolic factor within the gut–liver–kidney axis. Excess fructose that escapes small-intestinal metabolism induces epithelial stress, disrupts tight-junction proteins, and increases intestinal permeability, thereby facilitating the translocation of microbial products to the liver [165–167]. This gut-derived influx promotes hepatic lipogenesis, oxidative stress, and inflammation—key drivers of MASLD progression [165, 168]. Fructose-induced barrier dysfunction may further amplify systemic inflammation and contribute to kidney injury in metabolic disorders, underscoring the importance of dietary modulation in preserving gut–liver–kidney homeostasis [169–171].

Taken together, Figs. 2, 3 and 4 illustrate how individual microbial metabolites converge on shared mechanistic nodes to form a coordinated signaling network whose combined actions either sustain or disrupt axis homeostasis.

### Therapies based on the gut–liver–kidney axis

Tables 1 and 2 summarize current and emerging interventions targeting the gut–liver–kidney axis, providing an action-oriented view from mechanism to therapy. Although these approaches differ in their molecular targets, they ultimately converge on restoring metabolic balance, strengthening barrier function, and reducing inflammation and fibrosis. This mechanistic alignment offers a coherent basis for evaluating therapeutic potential.

**Table 1:** Therapeutic strategies targeting the gut–liver–kidney axis

Strategy category	Specific intervention	Disease type	Mechanism of action	Key evidence (Model/Clinical)	Article reference numbers
Microbiota modulation	*Lactobacillus*	CKD	Reduces TMA-producing bacteria; enhances gut barrier integrity; lowers TMAO accumulation	A systematic review from experimental studies	[172]
	*Bifidobacterium spp.*	ALD	Increases SCFA production; modulates host immunity; improves liver enzymes in alcoholic liver disease	Meta-analysis in ALD patients	[173, 174]
	*Akkermansia muciniphila*	CKD	Enhances gut barrier function; modulates glutamine metabolism; inhibits renal epithelial apoptosis	Preclinical CKD rat model	[175]
	Fecal microbiota transplantation	CKD	Reshapes microbial composition; reduces IS and PCS levels; expands SCFA-producing taxa; attenuates renal inflammation	6-month RCT in CKD patients; CKD rat models	[176, 177]
	Fecal microbiota transplantation	cirrhosis	Increases microbial diversity; reduces systemic inflammation; alleviates hepatic encephalopathy	A randomized clinical trial	[178–180]
Nutritional intervention	Low-protein diet (0.6–0.8 g/kg/day)	CKD	Lowers precursor load of uremic toxins (IS, PCS); reduces serum urea nitrogen	RCTs in CKD patients (Review)	[181]
	Low-protein diet + ketoanalogues	CKD	Maintains nitrogen balance; improves microbiota composition; enhances SCFA production	A systematic review and meta-analysis about CKD	[182, 183]
	Plant-based protein	CKD	Generates fewer sulfur-containing metabolites; increases dietary fiber for SCFA synthesis and barrier support	Comparative nutrition studies	[181]
	Soluble fiber (inulin)	CKD	Increases SCFA output; lowers IS/PCS; suppresses TLR4/NF-κB–mediated inflammation	A randomized controlled study	[184]
	Soluble fiber (guar gum)	MASLD	Promotes SCFA synthesis; strengthens gut barrier; reduces hepatic steatosis	MASLD mouse model	[185]
Physical adsorbents	AST-120	CKD	Adsorbs indole and p-cresol precursors in the gut; prevents systemic absorption of uremic toxins	Multicenter RCT in CKD	[186]
	MARS system	The acute-on-chronic and in the acute liver failure	Removes circulating bile acids, inflammatory mediators, and protein-bound toxins; improves hepatic and renal parameters	Clinical trials in liver failure and hepatorenal syndrome	[187, 188]

**Table 2:** Diet–microbiota–drug triad for precision modulation of the gut–liver–kidney axis

Component	Specific intervention	Functional role/mechanism	Supporting evidence & remarks	Study type &reference
Diet	Low-choline diet	Reduces substrate availability for microbial TMA formation, thereby lowering systemic TMAO	Epidemiological links to reduced plasma TMAO levels	Clinical research [143, 189, 190]
	High-fiber supplementation	Increases microbial SCFA production; strengthens gut barrier; suppresses TLR4/NF-κB inflammation	Riverw and RCT; high-fiber intake associated with ↓ hepatic fat content	Clinical research [191, 192]
	Plant-based protein sources	Generates fewer sulfur-containing uremic precursors; provides dietary fiber for SCFA synthesis	Comparative nutrition studies	Preclinical studies [181]
Microbiota	*Akkermansia muciniphila* (probiotic)	Enhances mucin layer and tight junctions; modulates glutamine metabolism	CKD rat model	Preclinical studies [175]
	*Roseburia spp.* (probiotic)	Boosts butyrate production; promotes regulatory T cell differentiation via HDAC inhibition	In vitro gut–liver axis model	Preclinical studies [23]
	Fecal microbiota transplantation (CKD)	Reshapes microbial composition; reduces IS and PCS levels; expands SCFA-producing taxa; attenuates renal inflammation	6-month RCT in CKD patients; CKD rat models	Clinical research and preclinical studies [176, 177]
	Fecal microbiota transplantation (cirrhosis)	Increases microbial diversity; reduces systemic inflammation; alleviates hepatic encephalopathy	A randomized clinical trial	Clinical research [178–180]
Therapy	AST-120 (oral adsorbent)	Adsorbs indole and p-cresol precursors in the gut; prevents systemic absorption of uremic toxins	Multicenter RCT in CKD	Clinical research [186]
	FXR agonist (e.g., obeticholic acid)	Activates FXR to restore bile acid homeostasis, suppress hepatic lipogenesis, and reduce fibrosis	Phase 1 clinical trials in NASH	Clinical research [193]
	FMO3 inhibitor	Inhibits hepatic conversion of TMA to TMAO, lowering circulating TMAO while preserving basal FMO3 function	Preclinical pharmacology studies in mice	Preclinical studies [194]
	MARS	Removes circulating bile acids, inflammatory mediators, and protein-bound toxins; improves hepatic and renal parameters	Clinical trials in liver failure and hepatorenal syndrome	Clinical research [187, 188]

Interventions are not isolated but operate in synergy through feedback loops. Evidence levels

● = preclinical or mechanistic studies

●● = small-scale or early-phase clinical trials

●●● = large-scale randomized controlled trials (RCTs) or guideline-supported meta-analyses

#### Microbiota modulation

In recent years, therapeutic strategies targeting the gut microbiota—such as probiotics and fecal microbiota transplantation (FMT)—have gained prominence as potential interventions to restore homeostasis within the gut–liver–kidney axis. Specific probiotic strains exert therapeutic effects by competitively inhibiting pathogenic bacteria, strengthening the intestinal barrier, and modulating host immune responses [195]. Preclinical studies provide compelling evidence supporting microbial interventions. In rodent models of CKD, FMT from healthy donors reshaped gut microbial composition and significantly suppressed tryptophan and lysine degradation pathways. This modulation reduced the production of protein-bound uremic toxins, such as IS and PCS, thereby attenuating renal injury [176]. Additionally, FMT promoted the recruitment of monocytic myeloid-derived suppressor cells, resulting in anti-inflammatory and anti-fibrotic effects in murine kidneys [196]. Colonization by *Akkermansia muciniphila*, induced by traditional medicine, further alleviated renal fibrosis by modulating glutamine metabolism and inhibiting apoptosis [175]. In liver disorders, probiotic interventions—particularly those involving *Bifidobacterium*—have shown promise in restoring microbial balance, enhancing intestinal barrier integrity, and regulating immune responses. A recent meta-analysis demonstrated that probiotic supplementation significantly improved liver function in patients with alcoholic liver disease, as evidenced by reductions in serum alanine aminotransferase, aspartate aminotransferase, and γ-glutamyl transferase (γ-GGT) levels [173]. Additional studies suggest that *Bifidobacterium* helps reestablish eubiosis and may serve as a safe adjunctive therapy in liver disease management [174].

Emerging clinical evidence in humans further supports microbiota-based interventions. A randomized, double-blind controlled trial in CKD patients reported that oral administration of FMT capsules for six months significantly slowed disease progression (13.3% vs. 53.8% in the placebo group) and stabilized renal function [177]. Microbial profiling revealed an increased abundance of anti-inflammatory taxa such as *Roseburia* species, suggesting that FMT may promote a protective microbial ecology. In patients with cirrhosis, FMT has also been shown to enhance microbial diversity, reduce systemic inflammation, and alleviate hepatic encephalopathy [178, 179].

Despite these encouraging results, several challenges remain. These include interindividual variability in donor microbiota, lack of standardized delivery methods, and the need for long-term safety data. Future directions should prioritize precision microbiota modulation guided by host genomics and metabolomics, with the goal of translating gut–liver–kidney axis manipulation into effective and personalized therapeutic regimens.

#### Bile acid–based therapeutic strategies

BA–targeted therapies have gained increasing attention as promising interventions for modulating gut–liver–kidney crosstalk. Obeticholic acid, a selective FXR agonist, has demonstrated efficacy in reducing liver fibrosis in patients with non-alcoholic steatohepatitis (NASH) [193]. Consistently, FXR-targeted agents can modulate bile-acid metabolism and ameliorate hepatic inflammation, although adverse effects and species-related differences in BA composition remain major barriers to clinical translation [69, 86, 197]. In the renal context, a novel polymer-based BA sequestrant capable of co-binding intestinal BAs and phosphate has shown promise in disrupting enterohepatic circulation and upregulating hepatic genes involved in cholesterol and BA biosynthesis. This approach improves glycemic control, lowers serum cholesterol, and slows diabetic kidney disease progression in type 2 diabetes animal models [198]. Despite these advances, interspecies differences and the complexity of BA regulatory networks highlight the need for more precise, axis-oriented BA-modulating strategies. Integrating BA-targeted approaches into gut–liver–kidney–axis–based therapy frameworks may help overcome current translational limitations and improve therapeutic outcomes.

#### Nutritional intervention

Dietary patterns and macronutrient intake—particularly protein and fiber—play a key role in modulating the gut–liver–kidney axis by influencing the gut microbiota and its metabolites. Several studies have shown that restricting protein intake to 0.6–0.8 g/kg/day significantly reduces serum urea nitrogen and levels of protein-bound uremic toxins such as IS and PCS, thereby slowing renal function decline [181, 199]. However, long-term adherence to low-protein diets may increase the risk of malnutrition and muscle wasting. To mitigate this, co-supplementation with ketoanalogues is recommended to maintain nitrogen balance, improve gut microbiota composition, and enhance SCFA production [182, 183]. Compared with animal protein, plant-based protein generates fewer proinflammatory sulfur-containing metabolites and is typically higher in dietary fiber, which supports SCFA synthesis and intestinal barrier integrity [181]. Although clinical data in MASLD remain limited, experimental models suggest that low-protein diets or selective amino acid restriction can suppress hepatic lipogenesis and inflammatory pathways, leading to improved liver histopathology [200].

Dietary fiber supplementation further promotes gut microbial fermentation and SCFA production, which in turn enhance intestinal barrier function, maintain immune homeostasis, and improve metabolic outcomes [191]. In a MASLD mouse model, the soluble fiber partially hydrolyzed guar gum ameliorated hepatic steatosis and inflammation by stimulating SCFA production, reinforcing gut barrier integrity, and inhibiting the TLR4/NF-κB signaling pathway [185]. SCFAs generated from various fiber types activate G protein–coupled receptors G protein-coupled receptor 41 and GPR43, regulating glucose and lipid metabolism and suppressing hepatic lipogenesis [201, 202]. Diets rich in whole grains, fruits, and vegetables have been associated with increased microbial diversity and SCFA output [191]. Clinical studies show that high fiber intake (≥ 25 g/day) correlates with reduced hepatic fat content and improved transaminase levels [191, 192]. In CKD, a randomized controlled trial demonstrated that combining a low-protein diet with inulin, a soluble fiber, for three months significantly reduced serum amine-bound uremic toxins and improved systemic inflammatory markers, highlighting the additive benefit of fiber in modulating uremic toxins and inflammation [184]. However, excessive and prolonged intake of fermentable fibers may alter bile acid metabolism and induce hepatic injury, underscoring the need for dose control [203, 204]. Together, the synergistic modulation of dietary protein and fiber offers a mechanistic rationale for gut–liver–kidney axis–targeted nutritional therapies in hepatic and renal disorders.

#### Physical adsorbent

Physical adsorbents interrupt the pathological cycle of the gut–liver–kidney axis via two principal mechanisms: (1) binding gut-derived toxin precursors in the intestinal lumen and (2) removing circulating middle-molecular-weight toxins through extracorporeal systems. AST-120, an oral carbon adsorbent composed of micron-sized spherical activated carbon particles, effectively binds uremic toxin precursors such as indole and p-hydroxyphenylacetic acid in the gastrointestinal tract. This prevents systemic absorption and promotes fecal excretion. A multicenter randomized controlled trial showed that AST-120 reduced the annual eGFR decline from approximately 15% to 12%, with greater renal protection observed in patients with marked proteinuria [186]. Beyond gastrointestinal adsorption, extracorporeal detoxification systems have been developed to target a broader spectrum of circulating toxins [205]. The molecular adsorbent recirculating system (MARS), a representative extracorporeal platform, integrates high-flux dialysis membranes with selective adsorbent circuits. This enables simultaneous removal of hydrophobic BAs, middle-molecular-weight inflammatory mediators, and protein-bound toxins. A 16-year single-center experience reported that MARS therapy improved clinical outcomes—including reduced serum bilirubin, hepatic encephalopathy, and pruritus—in patients with acute, chronic, and postoperative liver failure [187, 188]. In a prospective study of 10 patients with alcohol-related acute-on-chronic liver failure and type 1 hepatorenal syndrome, 41 MARS sessions resulted in mean reductions of 19%, 37%, 27%, and 14% in serum total bilirubin, ammonia, creatinine, and urea, respectively, with a 14-day survival rate of 90% [206]. Taken together, AST-120 and MARS offer complementary detoxification strategies. AST-120 primarily removes small-molecule toxin precursors in the gut, while MARS targets circulating middle-molecular-weight and protein-bound toxins. Their combined mechanisms may synergistically reduce systemic toxin burden and modulate the gut–liver–kidney axis both directly and indirectly [207, 208]. Future large-scale, multicenter randomized controlled trials in patients with CKD, MASLD, and hepatorenal syndrome are needed to establish the safety, timing, and potential for combination use of these therapies. A consolidated overview of these interventions is provided in Table 1, outlining their mechanisms of action and supporting evidence levels.

#### The diet–microbiota–drug triad: an integrated approach

Given the multi-organ and cross-system metabolic complexity of the gut–liver–kidney axis, therapeutic approaches must be equally multifaceted and synergistic. Emerging evidence supports a “diet–microbiota–pharmacotherapy” triad as an integrated regulatory strategy. As a foundational intervention, diet—via selective manipulation of dietary fiber, protein quality, and lipid composition—directly influences gut microbial composition and metabolic activity. The microbiota then acts as a central mediator, shaping the production of bioactive metabolites and modulating downstream signaling in the liver and kidneys. This microbial modulation can also enhance drug efficacy by altering local and systemic metabolic environments. Pharmacological agents for hepatic and renal diseases can further reinforce these effects at the molecular and cellular levels, complementing dietary and microbiota-based interventions. These components function within a dynamic regulatory feedback loop: diet serves as the input, the microbiota as the metabolic transformation hub, and drugs as the organ-specific effectors. When optimally coordinated, this triad may produce synergistic—or even multiplicative—therapeutic benefits.

However, empirical evidence demonstrating true mechanistic synergy among these components remains limited. Future research should prioritize intervention trials grounded in the diet–microbiota–pharmacotherapy model. Such studies should incorporate multi-omics profiling, multi-target analyses, and multi-endpoint clinical assessments. Moreover, advancing personalized nutrition, targeted microbiome modulation, and metabolism-directed drug combinations may facilitate more precise and effective therapies for gut–liver–kidney axis–related disorders. The diet–microbiota–drug triad offers a comprehensive framework for multi-level modulation of the gut–liver–kidney axis. When strategically integrated, the interactions among these components hold potential for substantial therapeutic synergy.

Overall, the therapeutic approaches summarized in Tables 1–2 converge on interrupting the pathological nodes outlined in Fig. 1 and the metabolite-specific pathways illustrated in Figs. 2, 3 and 4, thereby supporting mechanism-based interventions across the gut–liver–kidney axis.

### Challenges and the future

Despite significant advances in mechanistic understanding and translational applications related to the gut–liver–kidney axis, several critical, multidimensional challenges remain. One example is the dual role of TMAO. At physiological concentrations, it supports osmotic regulation; however, excessive levels contribute to renal interstitial fibrosis via NLRP3 inflammasome activation [209]. This has prompted interest in developing selective FMO3 inhibitors that lower renal TMAO accumulation without disrupting its physiological osmotic function [194]. Dietary interventions, though cost-effective and non-invasive, are often limited by poor long-term adherence. Factors such as the poor palatability of low-protein or high-fiber diets and their incompatibility with daily routines hinder compliance. Additionally, the lack of standardized dietary intake assessment tools in clinical practice presents a significant barrier. In a clinical trial involving patients with CKD, only ~ 50% of participants adhered to a prescribed low-protein diet (0.8 g/kg/day) throughout the intervention [210–212]. While psychological and behavioral support, as well as digital health technologies, show promise in enhancing adherence, high-quality evidence confirming their efficacy remains limited [213, 214]. FMT also poses safety and regulatory challenges. Current donor screening primarily relies on metagenomic diversity metrics, which do not fully eliminate the risk of latent pathogen transmission or immune incompatibility. A systematic review reported that 1.4% of FMT recipients experienced serious adverse events, including infections and death [215, 216]. Therefore, there is an urgent need to develop standardized safety protocols for synthetic microbial consortia and genetically engineered strains.

Pharmacological therapies are similarly constrained. For instance, although the FXR agonist obeticholic acid has shown efficacy in restoring bile acid homeostasis, its clinical use is limited by a high incidence of pruritus and elevated low-density lipoprotein cholesterol [217]. Further mechanistic studies and well-controlled trials are needed to evaluate alternative agents or combination strategies that reduce these adverse effects. Future research should focus on developing precise biomarkers that integrate metabolomics, metagenomics, and host genetic polymorphisms to guide individualized interventions. Multi-omics approaches, coupled with machine learning and artificial intelligence, may enable the rational design of personalized diet–microbiota–drug strategies. Additionally, the advancement of targeted drug delivery systems and next-generation microbial therapeutics should be evaluated in large-scale, multicenter, stratified randomized controlled trials. To ensure safe and sustainable clinical translation, robust ethical oversight and long-term surveillance frameworks are essential.

## Conclusion

**Fig. 5:**
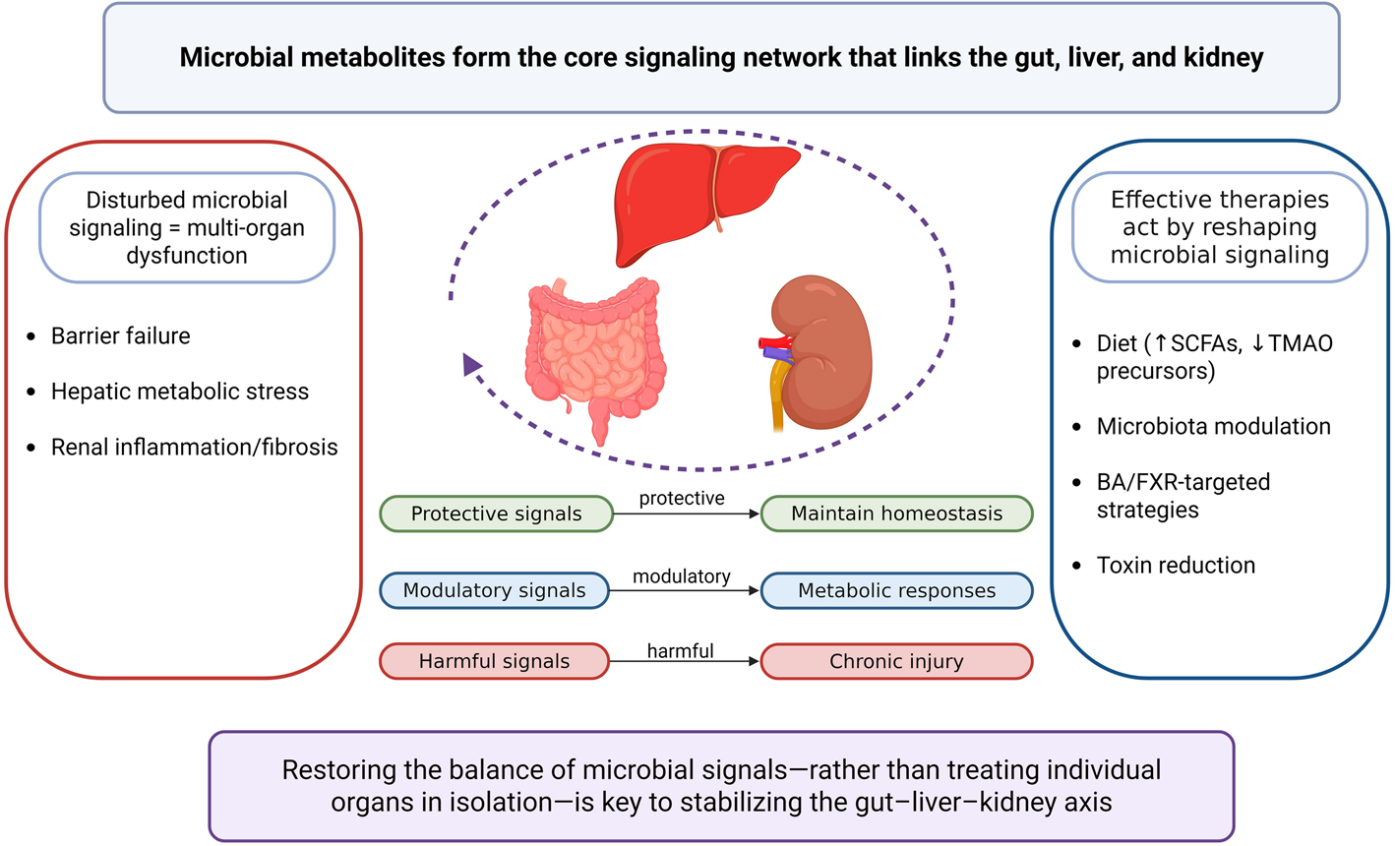
Microbial metabolites serve as integrators of gut–liver–kidney communication and central targets for therapeutic modulation

The gut–liver–kidney axis is central to maintaining metabolic and immune homeostasis through the action of gut-derived microbial metabolites. Disruption of this axis contributes to the pathogenesis of chronic liver and kidney diseases, with microbial dysbiosis, impaired intestinal barrier function, and systemic inflammation forming a self-perpetuating pathological cycle. As synthesized in Fig. 5, the pathophysiology of gut-liver-kidney axis disorders revolves around an imbalance in microbial metabolite signaling.

Current evidence underscores the therapeutic potential of restoring gut–organ communication to reverse disease progression and improve clinical outcomes. This review also highlights the diet–microbiota–drug triad as a conceptual framework for modulating the gut–liver–kidney axis. Dietary composition shapes microbial communities and their metabolic products; in turn, microbial activity influences drug metabolism and therapeutic efficacy, collectively impacting systemic physiology. Future efforts should focus on leveraging this triad to develop targeted, multi-organ treatment strategies. These insights underscore the necessity of integrated, multi-omics approaches for translating gut–liver–kidney axis mechanisms into personalized therapies. Progress in this field will depend on the integration of omics data, systems biology modeling, and rigorous clinical validation to design individualized interventions that restore inter-organ communication and metabolic balance.

## Data Availability

Not applicable. No datasets were generated or analyzed in this study.

## Abbreviations

AhR: Aryl hydrocarbon receptor
ALD: Alcohol-related liver disease
AMPK: AMP-activated protein kinase
Ang II: Angiotensin II
AST-120: Oral spherical carbon adsorbent AST-120
BAs: Bile acids
BCAA: Branched-chain amino acids
BSH: Bile salt hydrolase
CA: Cholic acid
CDCA: Chenodeoxycholic acid
CKD: Chronic kidney disease
CYP7A1: Cholesterol 7α-hydroxylase
CYP27A1: Cytochrome P450 family 27 subfamily A member 1
DCA: Deoxycholic acid
eGFR: Estimated glomerular filtration rate
FA: Fatty acid
FGF21: Fibroblast growth factor 21
FMO3: Flavin-containing monooxygenase 3
FMT: Fecal microbiota transplantation
FoxO1: Forkhead box protein O1
FXR: Farnesoid X receptor
GPR43: G protein-coupled receptor 43
HDAC: Histone deacetylase
LC3-II: Light chain 3-II
IDO1: Indoleamine 2,3-dioxygenase 1
IL‑1β: Interleukin-1 beta
IL‑18: Interleukin-18
ILC3: Lymphoid cell type 3
IPA: Indole-3-propionic acid
IS: Indoxyl sulfate
LPS: Lipopolysaccharide
MAFLD: Metabolic dysfunction-associated fatty liver disease
MARS: Molecular adsorbent recirculating system
MASLD: Metabolic dysfunction-associated steatotic liver disease
NAFLD: Non-alcoholic fatty liver disease
NASH: Non-alcoholic steatohepatitis
NF-κB: Nuclear factor-kappa B
NLRP3: NOD-like receptor family pyrin domain containing 3
PCS: P-cresyl sulfate
PERK: Protein kinase RNA-like endoplasmic reticulum kinase
PGC-1α: Proliferator-activated receptor gamma coactivator 1-alpha
PPARα: Peroxisome proliferator-activated receptor alpha
RAAS: Renin–angiotensin–aldosterone system
RCTs: Randomized controlled trials
SCFAs: Short-chain fatty acids
SREBP-1c: Sterol regulatory element-binding protein-1c
TCA: Taurocholic acid
TGF-β1: Transforming growth factor-beta 1
TGR5: Takeda G protein-coupled receptor 5
TLR4: Toll-like receptor 4
TMA: Trimethylamine
TMAO: Trimethylamine-N-oxide
ZO-1: Zonula occludens-1
7α-DH: 7α-Dehydroxylase
